# Global burden of chronic kidney disease attributable to hypertension due to high body mass index: projections to 2050

**DOI:** 10.1080/0886022X.2026.2751889

**Published:** 2026-11-02

**Authors:** Luohua Li, Guoqiang Zhu, Ke-Ke Wang, Sulier Maimaiti, Shun Wang, Chen Lu

**Affiliations:** Department of Nephrology Center, First Affiliated Hospital of Xinjiang Medical University, Urumqi, China

**Keywords:** Global burden of disease, high body mass index, chronic kidney disease due to hypertension, deaths, DALYs

## Abstract

Chronic kidney disease (CKD) affects approximately 9.1% of the global population, equivalent to approximately 843 million individuals as of 2021, with hypertension (HTN) as a major contributor. High body mass index (BMI) exacerbates HTN-related kidney injury, but the global burden of HTN-related CKD attributable to high BMI remains poorly characterized. Using data from the Global Burden of Disease (GBD) 2021 study for 1990–2021, we estimated attributable deaths, disability-adjusted life years (DALYs), the age-standardized mortality rate (ASMR), and the age-standardized disability-adjusted life-year rate (ASDR). Temporal trends were assessed using the estimated annual percentage change (EAPC), and a Bayesian age–period–cohort model was used to project rates to 2050. Attributable deaths increased from 36,476 in 1990 to 179,788 in 2021, and DALYs increased from 1.01 million to 4.26 million. ASMR increased from 1.09 to 2.19 per 100,000 (EAPC, 4.22%), and ASDR increased from 26.04 to 50.11 per 100,000 (EAPC, 3.63%). Central Sub-Saharan Africa had the highest ASMR (7.66 per 100,000), whereas High-income North America had the fastest increase in ASMR (EAPC, 5.63%). ASMR and ASDR were higher in men than in women. Death counts peaked at ages 85–89 years in both sexes, whereas DALYs peaked at ages 60–64 years in men and 65–69 years in women. Crude rates were higher in populations with a higher sociodemographic index (SDI), whereas the relative burden became more concentrated in lower-SDI populations; concentration indices changed from −0.10 to −0.17 for deaths and from −0.06 to −0.08 for DALYs. By 2050, ASMR is projected to reach 4.44 per 100,000 (95% credible interval [CI], 3.70–5.20), and ASDR is projected to reach 77.15 per 100,000 (95% CI, 1.81–152.49). The high BMI–attributable burden of HTN-related CKD is increasing and remains unequally distributed. These findings support integrated obesity prevention and hypertension control, with priority given to high-risk regions and older adults.

## 1. Introduction

Chronic kidney disease (CKD) is an increasingly important global public-health priority, contributing substantially to premature mortality, disability, and healthcare costs worldwide [1–3]. According to the GBD 2021 study, CKD affects an estimated 843 million people and ranks among the top causes of death and disability [2,3]. Hypertension is a leading etiologic factor for CKD, while elevated body-mass index (BMI ≥ 25 kg/m^2^) is a key modifiable driver that amplifies hypertension-related kidney injury *via* metabolic, inflammatory, and hemodynamic mechanisms [4–7].

Despite major advances in CKD epidemiology, the global burden of hypertension-related CKD (HTN-related CKD) that is specifically attributable to high BMI remains incompletely characterized. Previous GBD analyses have generally focused on overall CKD or on single risk factors, without explicitly quantifying the combined BMI → hypertension → CKD pathway or its long-term trajectory [1,8]. Using GBD 2021 data from 204 countries and territories, our study estimates deaths, disability-adjusted life years (DALYs), the age-standardized mortality rate (ASMR), and the age-standardized disability-adjusted life-year rate (ASDR) for HTN-related CKD attributable to high BMI from 1990 to 2021, examines patterns by age, sex, region, and sociodemographic index (SDI), and projects trends through 2050 using a Bayesian age–period–cohort (BAPC) model.

## 2. Materials and methods

### 2.1. Study design and data sources

This observational epidemiological analysis was conducted using data from the Global Burden of Disease 2021 (GBD 2021) study, coordinated by the Institute for Health Metrics and Evaluation (IHME) at the University of Washington. The GBD 2021 provides comprehensive estimates of the disease burden for 371 diseases and injuries, along with 88 risk factors, across 204 countries and territories from 1990 to 2021 [4,9].

Data on HTN-related CKD attributable to high BMI were retrieved using the Global Health Data Exchange (GHDx) query tool (http://ghdx.healthdata.org/gbd-results-tool). Extracted metrics included the numbers of deaths and DALYs, the age-standardized mortality rate (ASMR), and the age-standardized disability-adjusted life-year rate (ASDR). All estimates were stratified by sex, age group, calendar year, SDI quintiles, 21 GBD regions, and 204 countries or territories [9,10]. All reported values were accompanied by 95% uncertainty intervals (UIs), derived from 1,000 draws from the posterior distribution to account for both sampling and non-sampling error.

HTN-related CKD was defined according to the International Classification of Diseases, Tenth Revision (ICD-10) codes N18–N19 [3], encompassing structural or functional abnormalities of the kidney persisting for more than three months with potential health consequences. High BMI was defined, within the GBD framework, as a BMI ≥ 25 kg/m^2^ in adults aged ≥25 years, in accordance with the theoretical minimum risk exposure level (TMREL) [11]. The sociodemographic index (SDI) is a composite index ranging from 0 to 1, used to quantify the level of socioeconomic development based on lag-distributed income per capita, average years of education (≥15 years), and total fertility rate (<25 years) [12]. Countries were classified into five SDI quintiles: low (<0.46), low-middle (0.46–0.60), middle (0.61–0.70), high-middle (0.70–0.81), and high (>0.81) [13]. GBD regions were grouped according to epidemiological similarities and geographical proximity. As this study relied exclusively on publicly available, aggregated, and de-identified data, approval from an institutional review board and written informed consent were waived by the University of Washington. An overview of the study design and analytic workflow is provided in Figure 1.

**Figure 1.**
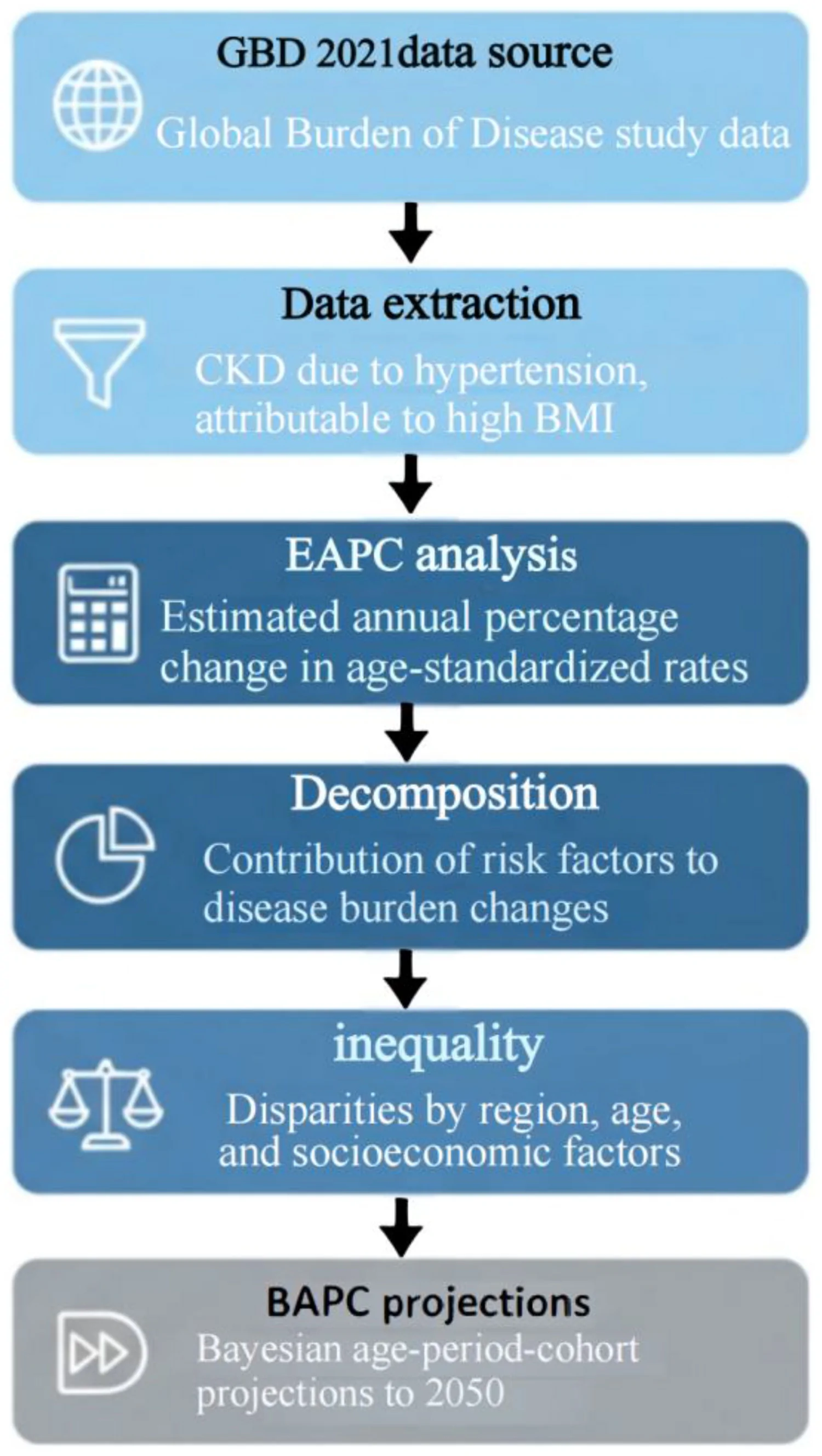
Overall study workflow for estimating and projecting the global burden of HTN-related CKD attributable to high BMI. The workflow covers data extraction from GBD 2021, temporal trend analysis using EAPC, decomposition analysis, health inequality analysis, and BAPC projections to 2050. Abbreviations: HTN, hypertension; CKD, chronic kidney disease; BMI, body mass index; GBD, Global Burden of Disease; EAPC, estimated annual percentage change; BAPC, Bayesian age–period–cohort.

### 2.2. Burden metrics and standardization

The disease burden was quantified using the numbers of deaths and DALYs, the latter being the sum of years of life lost (YLLs) due to premature mortality and years lived with disability (YLDs). YLLs were calculated as the product of the number of deaths and the standard life expectancy at the age of death. YLDs were computed by multiplying the disease prevalence by corresponding disability weights. Age-standardized rates (ASRs) were derived using the direct method, applying the GBD 2021 world standard population to permit valid comparisons across populations with varying age structures. The formula is expressed as:

$$\text{ASR}=\frac{\sum_{i=1}^{A}a_{i}w_{i}}{\sum_{i=1}^{A}w_{i}}\times 100,000$$

*α_i_*: the age-specific rates, *W_i_:* the number of persons (or weight) in the same age subgroup of the chosen reference standard population, A: the number of age groups, and *i*: the ith age class [14,15].

### 2.3. Temporal trend analysis

Temporal trends in the ASMR and the ASDR from 1990 to 2021 were summarized using the estimated annual percentage change (EAPC). EAPC represents the average annual percentage change in ASR over a specified period. We fitted a linear regression to the natural logarithm of the ASR as y = α + βx + ε, where y = ln(ASR) and x = calendar year. The EAPC was calculated using the formula: 100 × (exp(β)−1), with its 95% confidence interval (CI) also derived from the linear regression model [16,17]. An EAPC was interpreted as increasing if its estimate and the lower bound of its 95% CI were >0, as decreasing if the estimate and the upper bound of the 95% CI were <0, and otherwise as stable.

### 2.4. Decomposition analysis

To attribute changes in the absolute number of deaths and DALYs between 1990 and 2021 to demographic and rate-related drivers, we conducted a decomposition analysis partitioning the observed change into three components: population growth, population aging, and a rate-change component. The rate-change component reflects shifts in age-specific mortality and DALY rates independent of population size or age structure [18].

### 2.5. Health inequality analysis

Socioeconomic inequalities in burden were assessed using the slope index of inequality (SII) for absolute differences and the concentration index (CI) for relative differences. SII was estimated by weighted robust linear regression of ASMR or ASDR against countries’ relative SDI ranks (defined as the midpoint of the cumulative population distribution ordered by SDI). A positive SII indicates a higher absolute burden in higher-SDI populations. The CI was derived from the concentration curve, which plots the cumulative share of burden against the cumulative population ranked by SDI; CI values range from −1 to 1, with the sign indicating whether burden is concentrated among lower-SDI (negative) or higher-SDI (positive) populations.

### 2.6. Future projections

We applied a Bayesian age–period–cohort (BAPC) model to project ASMR and ASDR through 2050. In this framework, observed age-specific rates are modeled as a function of additive age, period, and cohort effects within a generalized linear modeling structure with a log link to ensure non-negative rates. Age, period, and cohort effects were specified as second-order random walks (RW2), which impose smoothness over adjacent categories while allowing flexible long-term trends. Posterior inference was obtained using integrated nested Laplace approximation (INLA) implemented in R, with default priors for the age, period, and cohort random effects. To enhance reproducibility, a fixed random seed (set to 12345) was used for all BAPC analyses. For each projected rate, we generated 1,000 posterior draws, and 95% credible intervals (CI) were defined as the 2.5th and 97.5th percentiles of the resulting posterior predictive distributions. Rates were modeled on the log scale and then back-transformed to the original per-100,000 scale, a procedure that can yield relatively wide CI when baseline rates are low.

### 2.7. Statistical analyses

All statistical analyses and data visualizations were performed using R software (version 4.4.1, https://www.r-project.org). The following packages were employed: dplyr, tidyr, stringr, and arrow for data processing and analysis; ggplot2, ggmap, rgdal, RColorBrewer, patchwork, and ggrepel for data visualization; and sf for geospatial analysis. Statistical significance was defined as *p* < 0.05 (two-tailed), with trends considered significant when the 95% CI excluded zero.

## 3. Results

### 3.1. Global burden of HTN-related CKD attributable to high BMI

Between 1990 and 2021, the global burden of HTN-related CKD attributable to high BMI increased markedly. In 2021, an estimated 179,788 deaths (95% UI: 91,324–260,252) were attributable to high BMI, compared with 36,476 deaths (95% UI: 17,465–59,219) in 1990—an increase of ≈392.9%. Over the same period, DALYs rose from 1,008,372 person-years (95% UI: 468,964–1,610,433) in 1990 to 4,257,037 person-years (95% UI: 2,119,854–6,136,203) in 2021, an increase of ≈322.2% (Supplementary Tables S1 and S2).

From 1990 to 2021, both the ASMR and the ASDR for HTN-related CKD attributable to high BMI approximately doubled globally, with consistently positive EAPCs across most regions and SDI groups. Detailed country- and region-specific estimates are provided in Supplementary Tables S1 and S2; Supplementary Figures S1; Figure 2.

**Figure 2.**
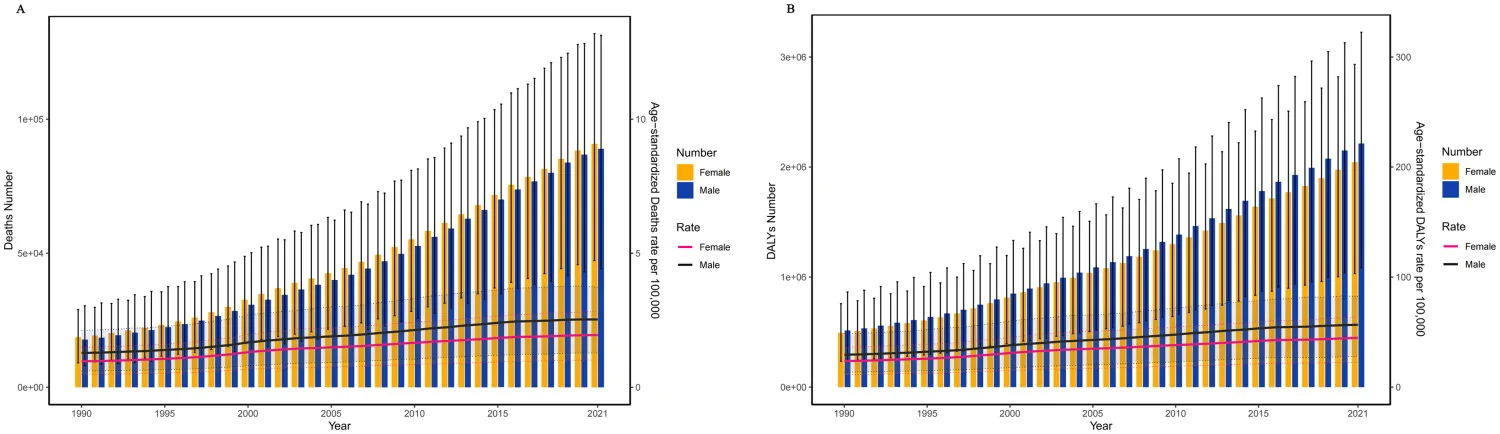
Global burden of HTN-related CKD attributable to high BMI by sex, 1990–2021. (A) Death counts and ASMR. (B) DALYs and ASDR. Bars show counts on the left axes, and lines show age-standardized rates on the right axes. Rates are expressed per 100,000 population. Vertical error bars show 95% UIs for the counts from GBD 2021. Abbreviations: HTN, hypertension; CKD, chronic kidney disease; BMI, body mass index; ASMR, age-standardized mortality rate; ASDR, age-standardized disability-adjusted life-year rate; DALYs, disability-adjusted life years; GBD, Global Burden of Disease; UI, uncertainty interval.

Overall, from 1990 to 2021, the global burden of HTN-related CKD attributable to high BMI showed a sustained and significant upward trend across absolute counts, DALYs, and age-standardized rates.

### 3.2. Regional burden of HTN-related CKD attributable to high BMI

From 1990 to 2021, both the ASMR and the ASDR for HTN-related CKD attributable to high BMI increased across all 21 GBD regions; EAPCs were positive in every region (Supplementary Tables S1 and S2).

In 2021, the highest ASMR was observed in Central Sub-Saharan Africa (7.66 per 100,000 population; 95% uncertainty interval [UI], 4.02–10.79), whereas the lowest was in Central Asia (0.31 per 100,000; 95% UI, 0.17–0.46). Over 1990–2021, ASMR rose most rapidly in High-income North America (EAPC 5.63%; 95% CI, 5.42–5.85) and slowest in Eastern Sub-Saharan Africa (EAPC, 1.02%; 95% CI, 0.78–1.26) (Supplementary Table S1).

For ASDR, the highest regional level in 2021 occurred in Southern Sub-Saharan Africa (154.33 per 100,000; 95% UI, 84.16–223.95) and the lowest in the High-income Asia Pacific (11.28 per 100,000; 95% UI, 5.40–18.34). From 1990 to 2021, ASDR increased fastest in High-income North America (EAPC, 4.91%; 95% CI, 4.71–5.11) and slowest in Eastern Sub-Saharan Africa (EAPC, 0.97%; 95% CI, 0.75–1.19) (Supplementary Table S2).

Between 1990 and 2021, ASMR and ASDR attributable to high BMI increased in all five SDI groups; all EAPCs were positive and statistically significant (*p* < 0.05) (Supplementary Tables S1 and S2). In 2021, the highest ASMR was in the Low–middle SDI group (2.46 per 100,000; 95% UI, 1.25–3.64) and the lowest in the High–middle SDI group (1.50 per 100,000; 95% UI, 0.75–2.23). Over 1990–2021, ASMR increased most rapidly in the High SDI group (EAPC, 5.44%; 95% CI, 5.23–5.64) and slowest in the Low SDI group (EAPC, 1.35%; 95% CI, 1.18–1.51) (Supplementary Table S1). For DALY-related metrics, the 2021 ASDR was highest in the Low–middle SDI group (59.68 per 100,000; 95% UI, 30.18–86.75) and lowest in the High–middle SDI group (32.26 per 100,000; 95% UI, 16.20–47.00). From 1990 to 2021, ASDR rose fastest in the High SDI group (EAPC, 4.49%; 95% CI, 4.33–4.65) and slowest in the Low SDI group (EAPC, 1.37%; 95% CI, 1.21–1.53) (Supplementary Table S2).

Overall, the growth in the high BMI–attributable burden of HTN-related CKD varied substantially across geographies, with particularly pronounced increases in High SDI regions and in High-income North America (Supplementary Tables S1 and S2; Figure 3).

**Figure 3.**
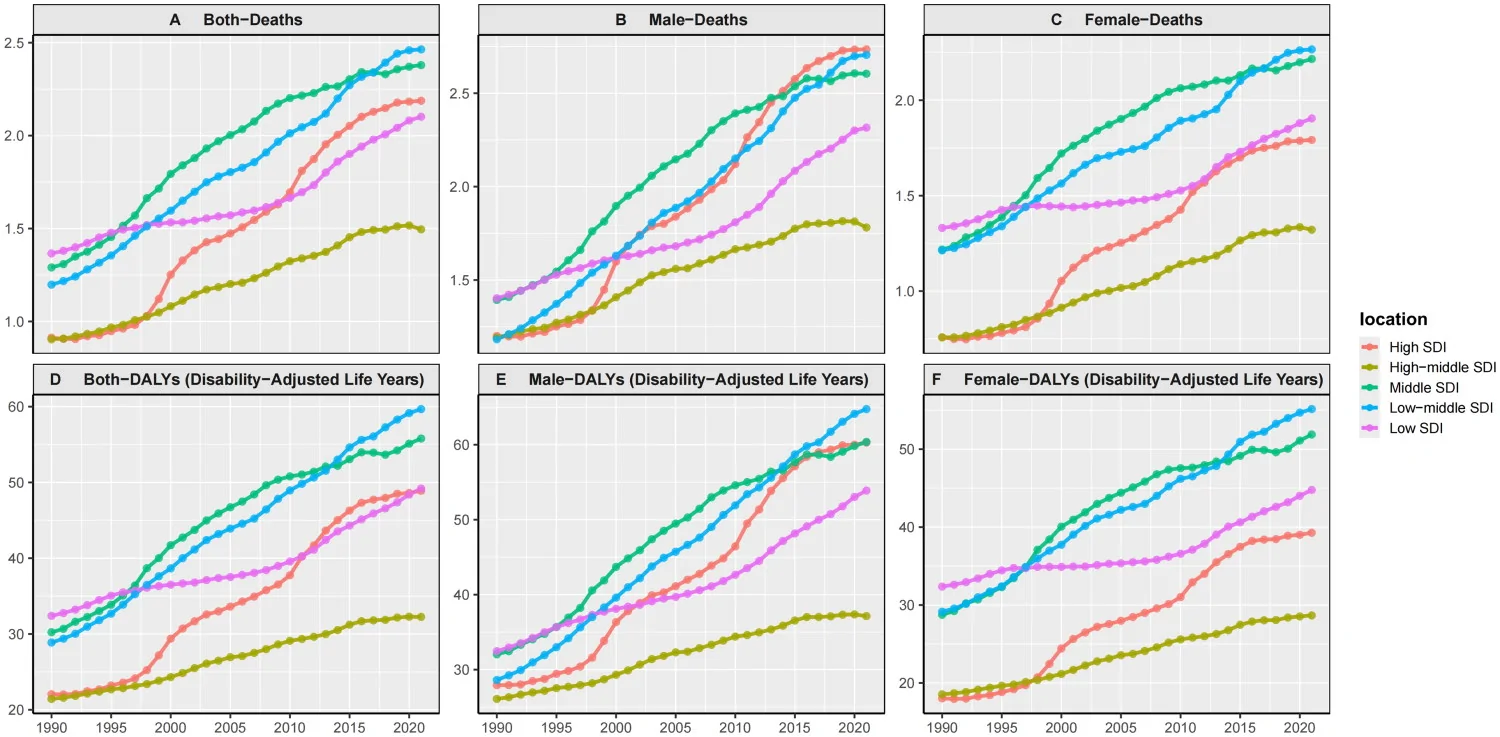
Age-standardized rates of HTN-related CKD attributable to high BMI by sex and SDI group, 1990–2021. ASMR is shown for (A) both sexes, (B) males, and (C) females. ASDR is shown for (D) both sexes, (E) males, and (F) females. Lines represent the high, high-middle, middle, low-middle, and low SDI groups. All rates are expressed per 100,000 population. Abbreviations: HTN, hypertension; CKD, chronic kidney disease; BMI, body mass index; SDI, sociodemographic index; ASMR, age-standardized mortality rate; ASDR, age-standardized disability-adjusted life-year rate; DALYs, disability-adjusted life years.

### 3.3. National burden of HTN-related CKD attributable to high BMI

From 1990 to 2021, most of the 204 countries analyzed experienced net increases in the burden of HTN-related CKD attributable to high BMI, with rises in both ASMR and ASDR in the majority of countries (Supplementary Table S3; Figure 4).

**Figure 4.**
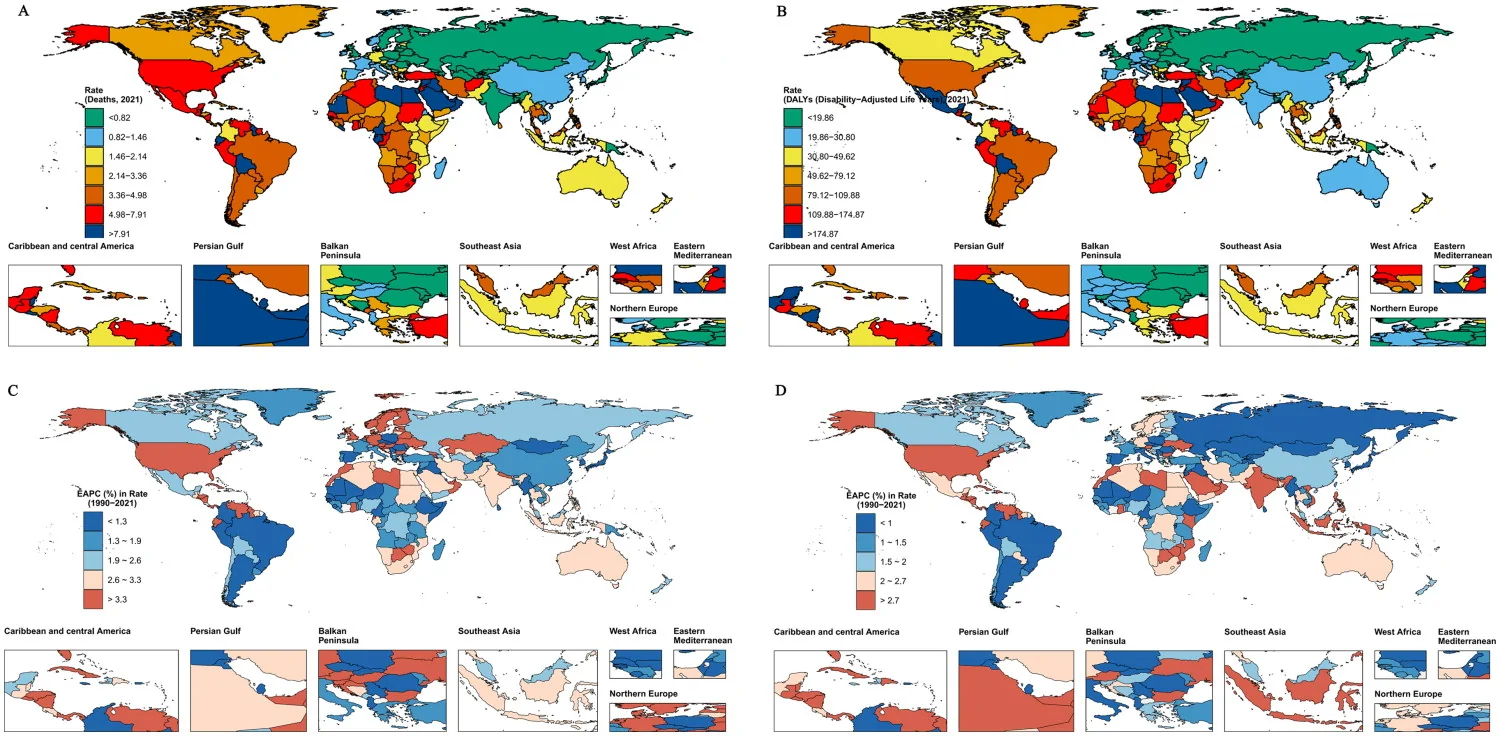
Geographical distribution and temporal trends of HTN-related CKD attributable to high BMI across 204 countries and territories. (A) ASMR in 2021. (B) ASDR in 2021. (C) EAPC in ASMR from 1990 to 2021. (D) EAPC in ASDR from 1990 to 2021. ASMR and ASDR are expressed per 100,000 population; EAPC is expressed as a percentage. Abbreviations: HTN, hypertension; CKD, chronic kidney disease; BMI, body mass index; ASMR, age-standardized mortality rate; ASDR, age-standardized disability-adjusted life-year rate; DALYs, disability-adjusted life years; EAPC, estimated annual percentage change.

In 2021, there was marked intercountry variation in ASMR. Saudi Arabia had the highest ASMR (17.747 per 100,000), followed by Egypt, whereas Tajikistan had the lowest ASMR (0.058 per 100,000) (Figure 4). Saudi Arabia also exhibited the largest upward ASMR trend between 1990 and 2021, while Finland showed the most pronounced decline (Figure 4).

A similar pattern was observed for DALYs: Saudi Arabia had the highest ASDR in 2021, followed by Egypt, and Tajikistan the lowest. Between 1990 and 2021, Saudi Arabia also had the fastest ASDR increase, whereas Finland again demonstrated the largest reduction (Figure 4).

### 3.4. HTN-related CKD burden attributable to high BMI based on sex and age

Global counts of deaths and DALYs attributable to high BMI-related CKD increased with age in 2021 (Figure 5A,B). Sex-specific analyses revealed distinct patterns: although absolute death counts among females were lower than among males in many age groups, males had higher absolute DALYs, ASMR, and ASDR overall. Both sexes experienced increases in burden from 1990 to 2021, with a more pronounced rise in males (Supplementary Tables S1 and S2; Figures 2 and 3).

**Figure 5.**
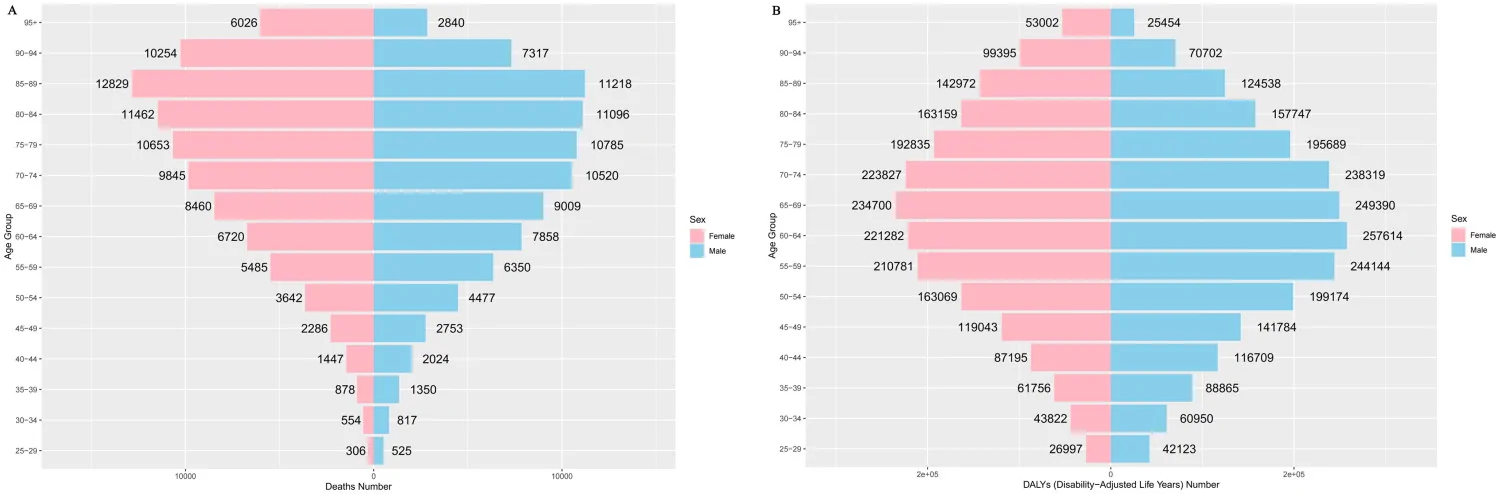
Age and sex distributions of the global burden of HTN-related CKD attributable to high BMI in 2021. (A) Death counts. (B) DALYs. Horizontal bars show the burden in each age group, with females on the left and males on the right. Abbreviations: HTN, hypertension; CKD, chronic kidney disease; BMI, body mass index; DALYs, disability-adjusted life years.

Age–sex–stratified peaks differed by outcome. In 2021, deaths peaked in the 85–89 age group for both females (11,218 deaths) and males (12,829 deaths) (Figure 5A). DALYs peaked at 65–69 years for females (234,700 DALYs) and at 60–64 years for males (257,614 DALYs) (Figure 5B).

Below age 65, female counts of deaths and DALYs attributable to high BMI were generally lower than male counts (e.g., ages 55–59: female deaths 5,485 vs male deaths 6,350; ages 45–49: female DALYs 119,043 vs male DALYs 141,784). Conversely, among those aged ≥65, the proportion of deaths and DALYs was higher in females than in males in several older age bands (e.g., ages 65–69: female deaths 9,009 vs male deaths 8,460; ages 80–84: female DALYs 163,159 vs male DALYs 157,747) (Figure 5A,B).

### 3.5. Attribution of high BMI

Figure 6 illustrates the contribution of high BMI to HTN-related CKD. Globally, high BMI accounted for 39.4% of deaths attributable to HTN-related CKD. Regional shares varied markedly: Southeast Asia had the lowest proportion (17.6%) while Australasia had the highest (63.2%) (Figure 6A). Similarly, high BMI explained 38.9% of DALYs globally, with Australasia contributing the largest share (66.2%) and Southeast Asia the smallest (17.2%) (Figure 6B).

**Figure 6.**
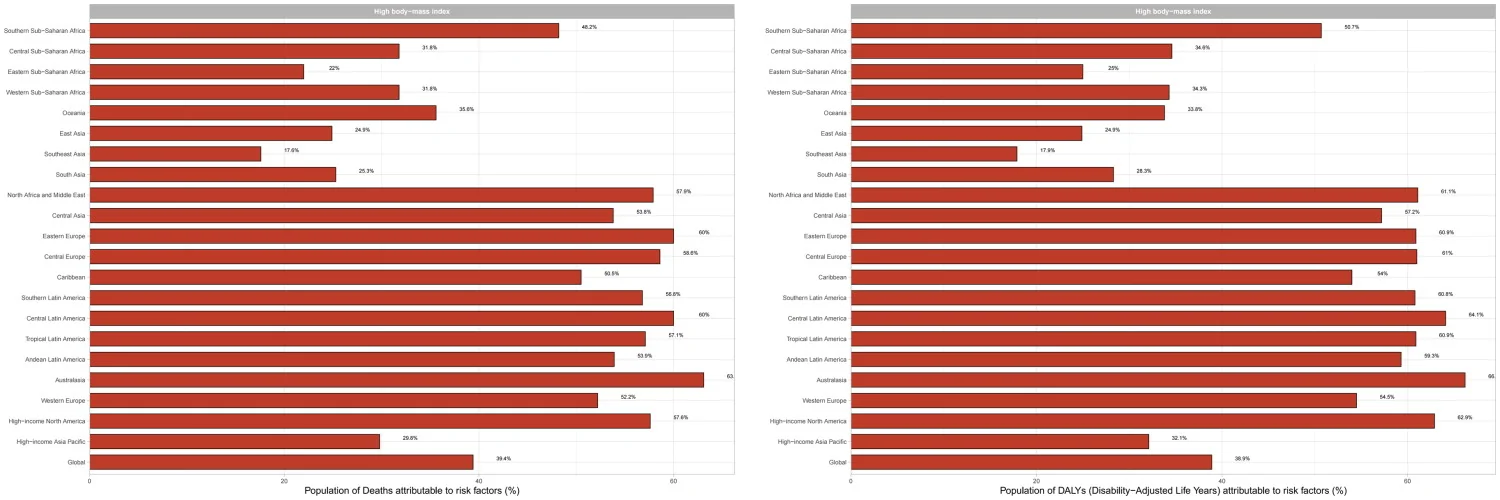
Proportions of HTN-related CKD deaths and DALYs attributable to high BMI in 2021. (A) Percentage of HTN-related CKD deaths attributable to high BMI. (B) Percentage of HTN-related CKD DALYs attributable to high BMI. Estimates are shown globally and for the 21 GBD regions. Abbreviations: HTN, hypertension; CKD, chronic kidney disease; BMI, body mass index; DALYs, disability-adjusted life years; GBD, Global Burden of Disease.

### 3.6. Global health inequality analysis in HTN-related CKD attributable to high BMI from 1990 to 2021

We examined absolute and relative inequalities by SDI. Compared with 1990, inequalities in deaths and DALYs attributable to HTN-related CKD and high BMI across SDI strata increased by 2021. Crude death rates generally rose with increasing SDI; the slope index of inequality (SII) for deaths was 2.19 (95% UI 1.28–3.10) in 2021 versus 0.27 (95% UI −0.05–0.58) in 1990 (Figure 7A). The SII for crude DALY rate also increased to 32.06 (95% UI 12.78–51.33) in 2021 from 5.88 (95% UI −1.36–13.11) in 1990 (Figure 7C).

**Figure 7.**
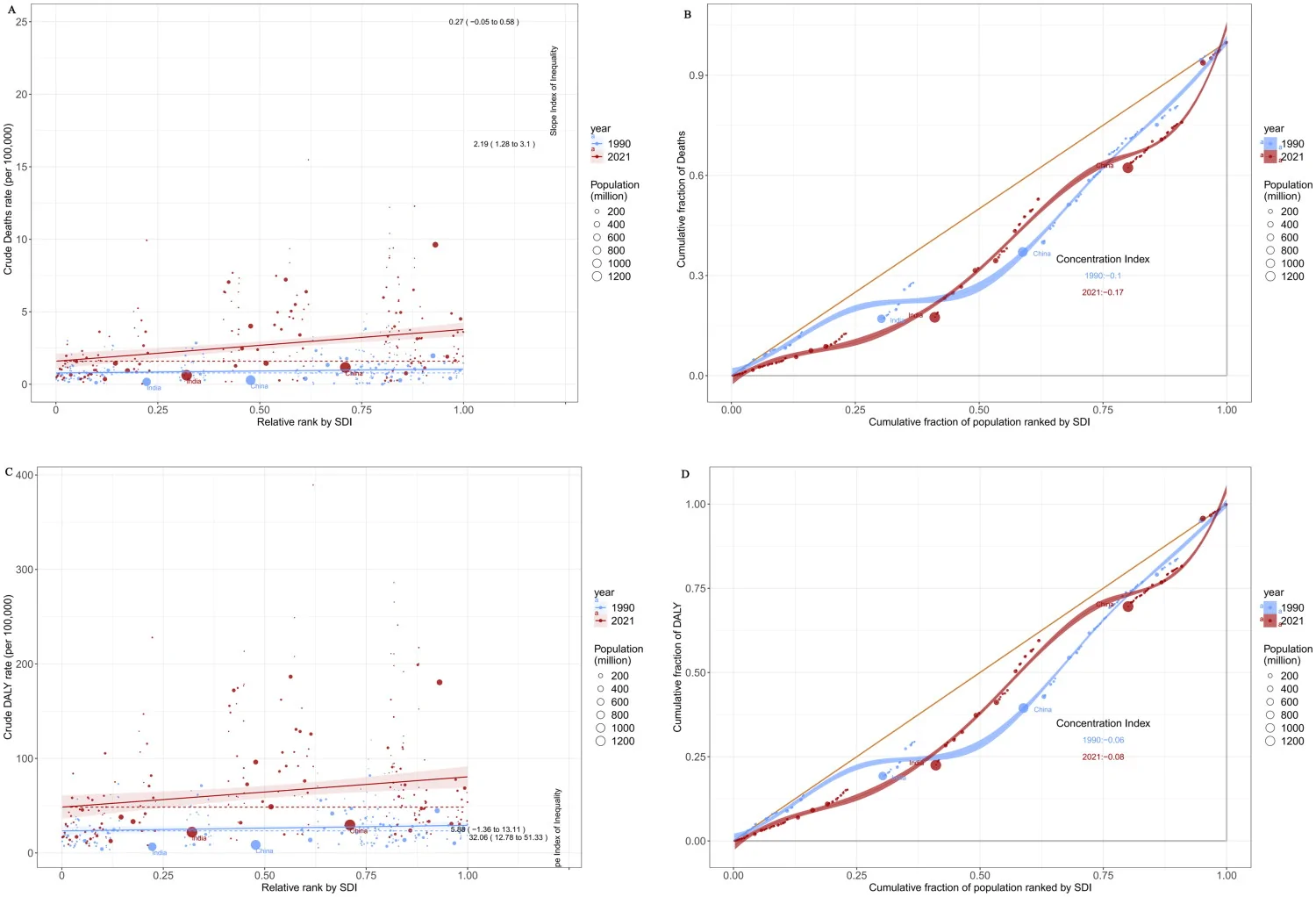
Socioeconomic inequalities in the burden of HTN-related CKD attributable to high BMI in 1990 and 2021, both sexes. (A) Crude death rates by relative SDI rank, with fitted relationships used to estimate the SII. (B) Concentration curves for deaths. (C) Crude DALY rates by relative SDI rank, with fitted relationships used to estimate the SII. (D) Concentration curves for DALYs. In panels A and C, circles represent countries, circle size indicates population, and rates are expressed per 100,000 population. In panels B and D, cumulative fractions of deaths or DALYs are plotted against cumulative population fractions ranked by SDI; the diagonal denotes equality. Abbreviations: HTN, hypertension; CKD, chronic kidney disease; BMI, body mass index; DALYs, disability-adjusted life years; SDI, sociodemographic index; SII, slope index of inequality.

The cumulative distribution curves for 1990 and 2021 diverged from the equality line, indicating that deaths became more concentrated among higher-SDI populations (Figure 7B). Concentration indices (CI) for deaths were −0.10 in 1990 and −0.17 in 2021, indicating that, over time, the relative burden of deaths has become more concentrated in lower-SDI populations. For DALYs, the CI shifted from −0.06 in 1990 to −0.08 in 2021, similarly suggesting a modest increase in the concentration of DALY burden among lower-SDI populations (Figure 7D).

### 3.7. Decomposition of changes in deaths and DALYs

To quantify the contributions of population growth, population aging, and epidemiological change to differences in deaths and DALYs between 1990 and 2021, we performed decomposition analyses (Figure 8A–D; Supplementary Table S3). Globally, deaths from HTN-related CKD attributable to high BMI increased by 849,607 and DALYs increased by 11,148,370 over the study period. Epidemiological change (i.e., changes in age-specific rates) was the largest contributor to the increase in deaths (48.34%), followed by population growth (26.82%) and population aging (24.84%). For DALYs, epidemiological change accounted for 49.05% of the increase, population growth for 29.90%, and aging for 21.04%.

**Figure 8.**
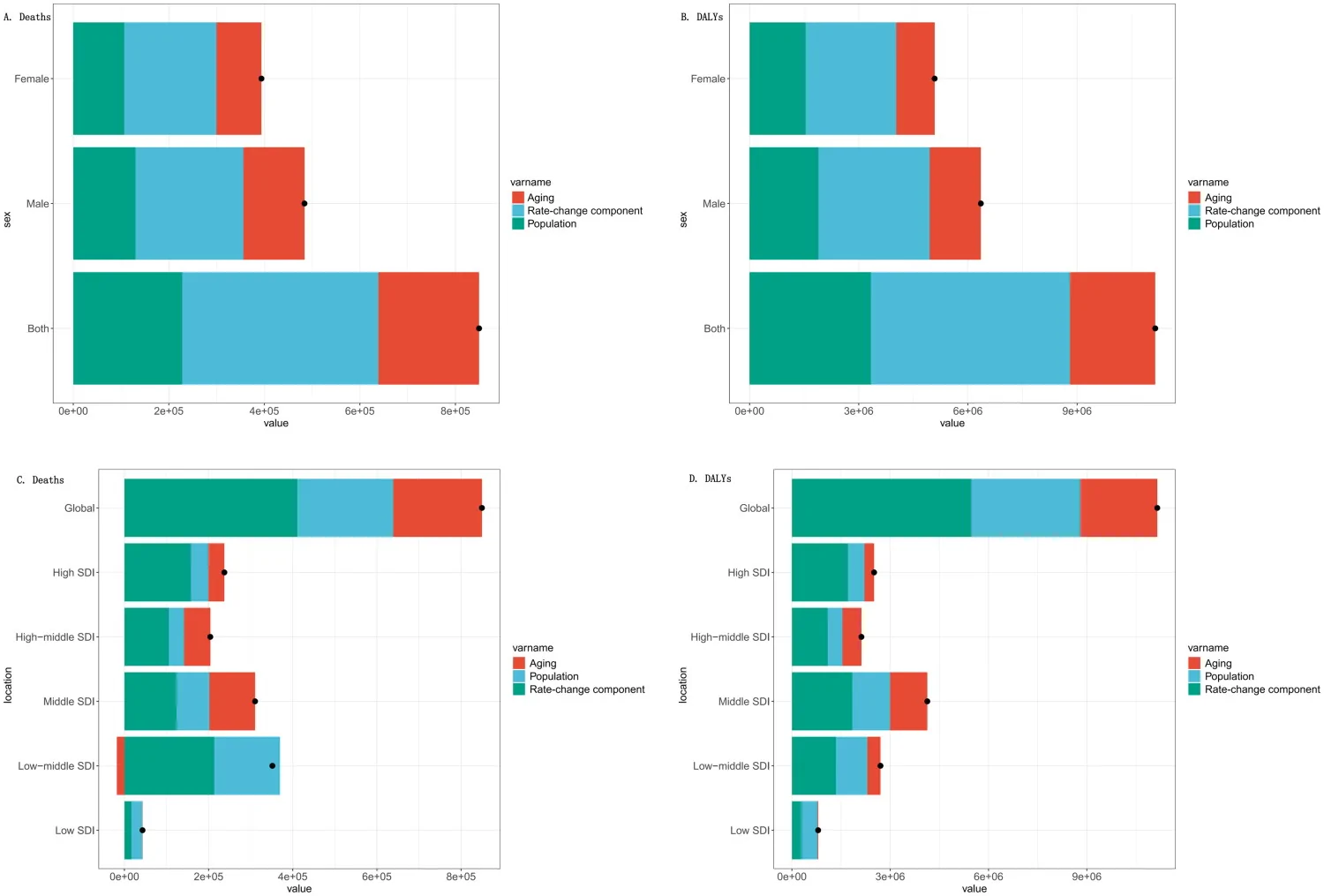
Decomposition of changes in the burden of HTN-related CKD attributable to high BMI, 1990–2021. Contributions of population growth, population aging, and changes in age-specific rates are shown for (A) deaths by sex, (B) DALYs by sex, (C) deaths globally and by SDI group, and (D) DALYs globally and by SDI group. Bar segments represent the three components, and black dots indicate the net change. Abbreviations: HTN, hypertension; CKD, chronic kidney disease; BMI, body mass index; DALYs, disability-adjusted life years; SDI, sociodemographic index.

Sex-specific decomposition showed broadly similar patterns. For deaths, epidemiological change explained 46.61% of the increase in males and 48.94% in females, whereas population growth contributed 26.95% and 27.08%, and aging contributed 26.44% and 23.98%, respectively. For DALYs, the corresponding contributions of epidemiological change were 48.10% in males and 48.96% in females, with population growth accounting for 29.75% and 30.30% and aging for 22.15% and 20.75%, respectively. Thus, in both sexes, changes in age-specific rates were the dominant driver, while demographic factors each contributed roughly one quarter of the observed increases.

By SDI strata, epidemiological change remained the principal contributor to increases in deaths across most development levels, particularly in high-SDI regions where it accounted for 66.44% of the increase (population growth 17.55%, aging 16.01%). In high-middle, middle, and low-middle SDI regions, epidemiological change explained 51.64%, 39.73%, and 60.64% of the increase in deaths, respectively. In low-SDI regions, epidemiological change and population growth contributed 38.48% and 60.19% of the increase, while aging had only a minor effect (1.32%). For DALYs, epidemiological change similarly predominated in high-, high-middle, middle-, and low-middle SDI strata (68.13%, 51.47%, 44.58%, and 49.61%, respectively), whereas in low-SDI regions population growth was the leading driver (60.88% vs 35.87% for epidemiological change). Overall, these decomposition results indicate that changes in age-specific rates—reflecting shifts in epidemiological profiles and risk-factor exposure—rather than demographic dynamics alone, largely explain the global and regional rises in HTN-related CKD burden attributable to high BMI, although population growth remains a major driver in the lowest-SDI settings and aging exerts a modest, context-specific impact.

### 3.8. BAPC projections of global HTN-related CKD burden attributable to high BMI (2022–2050)

Using BAPC models to project 2022–2050 trajectories, both global ASMR and ASDR attributable to high BMI are predicted to continue rising (Figure 9A,B). By 2050, the projected global ASMR (4.44 per 100,000; 95% CI: 3.70–5.20) is approximately double the 2021 level (2.19 per 100,000), and the projected ASDR (77.15 per 100,000; 95% CI: 1.81–152.49) is roughly 50% higher than in 2021 (50.11 per 100,000). These projections thus suggest a continued substantial rise in the BMI-attributable HTN-related CKD burden under a ‘business-as-usual’ scenario. The wide 95% CI, particularly at later time points, indicate substantial model and data uncertainty and should be interpreted as ranges of plausible future trajectories rather than precise forecasts.

**Figure 9.**
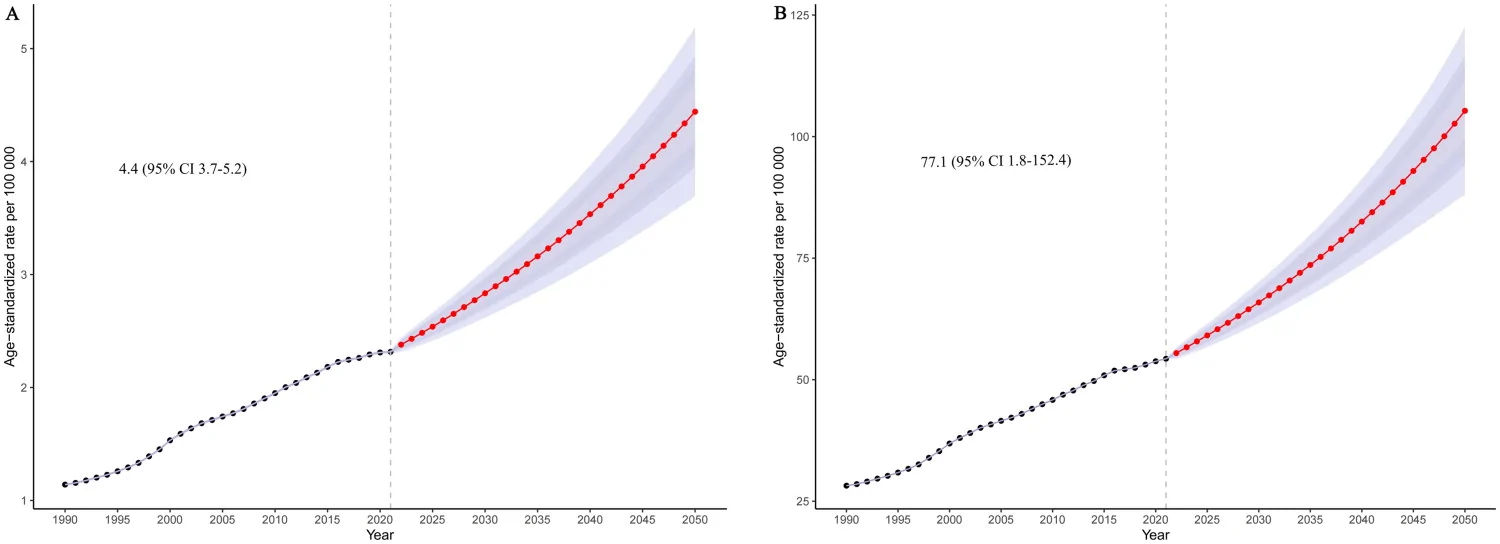
Historical estimates and projections of the global burden of HTN-related CKD attributable to high BMI, 1990–2050. (A) ASMR. (B) ASDR. Historical estimates cover 1990–2021, and BAPC projections cover 2022–2050. Projected point estimates are posterior medians, and shaded bands represent 95% CIs. All rates are expressed per 100,000 population. Abbreviations: HTN, hypertension; CKD, chronic kidney disease; BMI, body mass index; ASMR, age-standardized mortality rate; ASDR, age-standardized disability-adjusted life-year rate; DALYs, disability-adjusted life years; BAPC, Bayesian age–period–cohort; CI, credible interval.

## 4. Discussion

### 4.1. Key findings

This study provides the first comprehensive quantification of the global burden of HTN-related CKD that is attributable to high BMI. Uniquely, we focused on the BMI → hypertension → CKD causal pathway and used BAPC projections to 2050, extending prior GBD analyses that evaluated overall CKD or single risk factors without isolating this combined etiologic sequence. Over the past three decades (1990–2021) we observed a marked and sustained increase: deaths attributable to high-BMI exacerbated HTN-related CKD rose nearly fourfold and DALYs roughly threefold, substantially outpacing the more gradual rise in overall CKD burden over the same period. Consequently, high BMI emerged as a major contributor to CKD outcomes in 2021. The burden is ubiquitous—affecting all GBD regions and both sexes—but shows important geographic and sex-specific heterogeneity, with especially steep relative increases in higher-SDI regions (for example, North America and high-income Asia-Pacific) while traditionally high-burden regions (such as sub-Saharan Africa and South Asia) continue to bear the greatest absolute rates. Men had higher ASMR and ASDR and a faster rise than women; however, in advanced age groups older women approached or exceeded men in absolute deaths and DALYs, even though ASR remained higher in men. Projections indicate that, absent effective interventions, attributable mortality and DALY rates will approximately double again by 2050. These results highlight a rapidly accelerating global health challenge at the intersection of obesity, hypertension, and kidney disease [2,19].

### 4.2. Mechanistic interpretation

The pronounced upward trajectory likely reflects the synergistic interaction between the global obesity epidemic and suboptimal control of cardiometabolic risk factors. Excess adiposity biologically amplifies hypertension’s harmful effects on the kidney *via* multiple mechanisms—including activation of the renin–angiotensin–aldosterone system, persistent low-grade inflammation, insulin resistance, and glomerular hyperfiltration—which together accelerate renal injury [20]. The near 392% increase in deaths attributable to high-BMI, hypertension-driven CKD since 1990 parallels the dramatic global rise in obesity prevalence. In this context, our findings align with global risk assessments that identify high BMI among leading contributors to CKD mortality alongside hypertension and hyperglycemia [7], and indicate that obesity is shifting the etiologic landscape of CKD rather than merely co-occurring with existing trends [21].

### 4.3. Sex and regional inequities

Sex differences in burden warrant careful consideration. Men experienced higher age-standardized death and DALY rates from high-BMI–related HTN-related CKD and a slightly more rapid temporal increase. This pattern probably reflects higher baseline hypertension prevalence, greater visceral adiposity, and differing risk behaviors in men, as noted by prior studies [6] Nonetheless, women—particularly at older ages—carry an equal or greater absolute CKD burden, which is consistent with women’s longer life expectancy and higher CKD prevalence in later life. Differential access to care and sociocultural determinants may further modulate these patterns in some regions. Overall, the data imply that interventions must be inclusive and sex-sensitive: for example, intensified risk-factor control and early screening in younger obese men, and targeted case-finding and management in older women.

Our results corroborate and extend earlier GBD and epidemiologic analyses that documented a growing global CKD burden and emphasized metabolic risk factors as central drivers [6,7]. Previous GBD reports often assessed CKD burden or risk factors individually; by isolating the intersection of obesity → → hypertension → CKD, we quantify a specific and rapidly rising etiologic pathway that prior aggregated analyses did not explicitly separate [2,6]. Using the updated GBD 2021 dataset and producing forecasts to 2050, our study provides a more current and forward-looking assessment than earlier projections limited to the 2020s–2030s [22,23]. Notably, high-income North America showed one of the fastest relative increases (EAPC for ASMR ≈ 5.6%/yr, 1990–2021), a pattern compatible with other recent GBD-based findings that revealed rapid burden growth in some higher-SDI settings while persistent absolute burdens remain in low-SDI regions [24,25].

### 4.4. Clinical and policy implications

Building on these mechanistic and distributional patterns, several clinical and policy implications emerge. These findings have several practical implications. Mechanistically, the pronounced synergy between obesity and hypertension implies that even modest improvements in BMI or blood-pressure control may yield disproportionate benefits for renal outcomes by interrupting the obesity-driven hypertensive injury cascade [26]. Clinically, our results support integrating weight management into hypertension care pathways (including dietary sodium reduction and structured weight-loss programs) and prioritizing routine kidney function and proteinuria screening among hypertensive individuals with high BMI, so that reno-protective therapies (e.g., RAAS blockade, sodium-glucose co-transporter 2 (SGLT2) inhibitors where indicated) can be initiated earlier [27–30]. From a health-systems perspective, high-SDI regions facing aging populations and long-standing obesity epidemics should prepare for increasing numbers of advanced CKD and dialysis patients, and concurrently invest in upstream obesity prevention strategies (such as food-environment regulation, sugar-sweetened-beverage taxation, and urban designs that promote physical activity) to avert future demand on specialized renal services.

For low- and middle-SDI regions, the situation is compounded by a double burden of disease: persistent infectious and undernutrition challenges alongside rising obesity and cardiovascular risks. Many such countries exhibit low hypertension awareness and control, which amplifies the renal impact of rising BMI; our data therefore support urgent strengthening of primary-care systems for early hypertension detection and treatment and culturally tailored health-education interventions to curb unhealthy diets and sedentary behaviors [31–33]. International funding and technical cooperation may be required to support scalable prevention and treatment programs in resource-limited settings.

At the policy level, CKD should be elevated within national and international NCD agendas to match the priority given to cardiovascular disease, cancer, diabetes, and chronic respiratory disease [34,35]. Quantifying the attributable burden from obesity provides an evidence base for cost-effectiveness and budget-impact analyses that can inform resource allocation—for example, investments in community weight-management programs, CKD screening among high-risk groups, and workforce planning for nephrology services. These regional patterns and corresponding policy options are summarized in Table S4, which highlights potential levers—such as obesity control, hypertension screening, and CKD awareness campaigns—that could plausibly attenuate the projected rise in DALYs.

### 4.5. Limitations and future directions

This analysis has several limitations. First, it is a secondary analysis of GBD 2021 data, which synthesizes heterogeneous epidemiologic sources; in many low-SDI settings vital registration and CKD surveillance are incomplete, requiring model-based estimation and increasing uncertainty—hence country-level estimates with sparse input data should be interpreted cautiously [1]. Second, GBD cause attribution relies on modeling of causes of death and comparative risk assessment; ‘HTN-related CKD’ and the fraction attributable to high BMI are therefore population-level constructs that may misclassify individuals with multiple coexisting risks (for example, obesity plus diabetes). Because GBD risk factors are modeled independently as risk–outcome pairs, attributable burdens are not mutually exclusive and may overlap (for example, obesity and hyperglycemia both acting *via* hypertension), so the true joint contribution of clustered risks may be smaller than the sum of individual risk-specific estimates. Consistent with this structure, the GBD comparative risk-assessment framework assumes independence in risk attribution and uses a theoretical minimum risk exposure distribution (BMI 20–25 kg/m^2^), implying that risk-specific estimates should be interpreted as approximations of complex, overlapping causal pathways rather than as simple additive contributions [36,37]. By focusing on the BMI-attributable fraction within hypertension-attributed CKD we capture a defined causal pathway but likely provide a conservative estimate of obesity’s total contribution (which also operates *via* diabetes and other mechanisms). Third, our BAPC projections assume that recent trends in age-, period-, and cohort-specific rates continue into the future. They do not explicitly incorporate potential structural changes such as widespread adoption of potent anti-obesity medications, major policy shifts, or unforeseen shocks (e.g., pandemics), any of which could substantially alter future trajectories. The relatively wide CI around 2050 projections, particularly for ASDR, reflect both this structural uncertainty and the data limitations described above. Although out-of-sample validation suggests that the model reproduces recent trends reasonably well, projections should be interpreted as plausible scenarios rather than precise forecasts. Given that our BAPC model focused on age-standardized rates rather than explicit population counts, we report relative percentage changes in ASMR and ASDR rather than absolute projected deaths and DALYs, and future work should integrate formal population projections to estimate absolute case numbers. Fourth, we did not perform formal out-of-sample validation (for example, training the BAPC model up to 2016 and predicting 2017–2021 with RMSE or other goodness-of-fit metrics), so the predictive performance of our forecasts has not been quantitatively assessed and should be interpreted with caution. Finally, we did not examine within-country heterogeneity (urban-rural, socioeconomic, or racial/ethnic disparities), which are important determinants of CKD risk and service access and merit targeted national-level investigation [38].

Despite these limitations, the study has strengths: a standardized, comparable analytic framework applied across 204 countries over *a* > 30-year interval strengthens confidence in the global and regional inferences. Our explicit focus on the obesity → hypertension → CKD pathway fills a critical evidence gap and yields policy-relevant, actionable insights.

In summary, the convergence of the obesity pandemic and hypertension has generated a substantial and accelerating global burden of CKD, which is projected to worsen by 2050 absent effective interventions. By quantifying the contribution of high BMI to HTN-related CKD, this work identifies a major, modifiable driver of kidney disease and underscores the need for integrated prevention and treatment strategies. Immediate priorities include: (1) integrating weight-management interventions into hypertension care; (2) improving early detection of kidney impairment among obese hypertensive individuals; (3) prioritizing upstream public-health measures to curb obesity; and (4) strengthening primary-care systems in low-resource settings. Future research should evaluate implementation strategies (including the renal effects of emerging anti-obesity therapies such as glucagon-like peptide-1 receptor agonists), generate more granular subnational data to inform targeted interventions, and develop scenario-based forecasting that models different obesity-control trajectories. Although our projections are descriptive rather than intervention-model-based, they underscore that preventive strategies targeting obesity and hypertension could plausibly reduce the future global burden of CKD.

## Supplementary Material

Supplemental Material

Supplemental Material

Supplemental Material

Supplemental Material

Supplemental Material

Supplemental Material

## Data Availability

The data that support the findings of this study are available in the Global Health Data Exchange (GHDx) at https://ghdx.healthdata.org/gbd-2021, reference number [4,39]. These data were derived from the following resources available in the public domain: GBD 2021 study data, which can be accessed *via* the GHDx query tool (http://ghdx.healthdata.org/gbd-results-tool).
