# Supplementary material for: Global burden of chronic kidney disease attributable to hypertension due to high body mass index: projections to 2050
Source: Ren Fail. 2026 Nov 2;48(1):2751889. doi: 10.1080/0886022X.2026.2751889 (PMC13729631; doi:10.1080/0886022X.2026.2751889)
Supplement: Supplementary file 1 [file IRNF_A_2751889_SM4225.docx]

**Table 1** Deaths and ASMR of HTN-related CKD burden attributable to High body-mass index in 1990 and 2021 and the temporal trends from 1990 to 2021

| Characteristics | Death cases, No. (95% *UI*) | | ASMR per 100,000 people (95% *UI*) | | EAPC of ASMR, % (95% *CI*) |
| --- | --- | --- | --- | --- | --- |
|  | 1990 | 2021 | 1990 | 2021 | 1990–2021 |
| **Global** | 36476.31 (17465.27, 59218.78) | 179788.28 (91323.55, 260251.54) | 1.09 (0.52, 1.74) | 2.19 (1.11, 3.19) | 4.22 (4.13,4.32) |
| **Sex** |  |  |  |  |  |
| Male | 17804.54 (8037.14, 30385.89) | 88940.88 (44215.88, 131307.73) | 1.28 (0.6, 2.11) | 2.53 (1.27, 3.73) | 4.29 (4.21,4.37) |
| Female | 18671.77 (9115.04, 28969.76) | 90847.4 (47294.65, 131912.75) | 0.97 (0.48, 1.49) | 1.95 (1.02, 2.82) | 4.16 (4.04,4.27) |
| **SDI** |  |  |  |  |  |
| Low SDI | 2580.53 (1175.1, 4492.49) | 9007.52 (4323.97, 15139.22) | 1.37 (0.61, 2.4) | 2.1 (1, 3.49) | 1.35 (1.18,1.51) |
| Low-middle SDI | 6120.92 (2850.96, 10374.36) | 31606.43 (16126.64, 46554.49) | 1.2 (0.55, 2.02) | 2.46 (1.25, 3.64) | 3.83 (3.78,3.88) |
| Middle SDI | 10525.42 (4690.01, 17535.22) | 57611.99 (27791.57, 87626.1) | 1.29 (0.59, 2.15) | 2.38 (1.18, 3.65) | 4.57 (4.40,4.74) |
| High-middle SDI | 7334.17 (3610.45, 11664.84) | 28393.77 (14297.86, 42276.82) | 0.9 (0.45, 1.41) | 1.5 (0.75, 2.23) | 4.02 (3.94,4.11) |
| High SDI | 9868.52 (4969.88, 15205.51) | 52995.27 (27300.94, 74271.02) | 0.91 (0.46, 1.4) | 2.19 (1.16, 2.99) | 5.44 (5.23,5.64) |
| **GBD region** |  |  |  |  |  |
| Andean Latin America | 679.83 (277.81, 1107.03) | 3897.33 (1880.25, 5637.57) | 3.58 (1.45, 5.88) | 6.83 (3.28, 9.91) | 4.18 (3.76,4.60) |
| Australasia | 169.25 (93.89, 248.75) | 980.09 (520.21, 1406.62) | 0.8 (0.44, 1.16) | 1.5 (0.8, 2.15) | 4.99 (4.54,5.43) |
| Caribbean | 501.62 (248.89, 767.7) | 2113.07 (1128.71, 3028.23) | 2.08 (1.02, 3.2) | 3.88 (2.07, 5.57) | 4.21 (4.06,4.35) |
| Central Asia | 47.85 (26.07, 74.08) | 232.67 (133.81, 338.42) | 0.1 (0.06, 0.17) | 0.31 (0.17, 0.46) | 3.63 (3.10,4.16) |
| Central Europe | 1134.77 (652.4, 1648.35) | 2418.64 (1441.96, 3399.52) | 0.84 (0.5, 1.2) | 1.02 (0.61, 1.43) | 3.78 (3.42,4.13) |
| Central Latin America | 2263.81 (1159.93, 3323.98) | 14493.86 (7951.48, 20177.36) | 3.14 (1.64, 4.68) | 5.94 (3.27, 8.26) | 5.16 (4.63,5.69) |
| Central Sub-Saharan Africa | 440.28 (205.84, 771.65) | 2060.03 (959.52, 3520.95) | 2.52 (1.19, 4.5) | 4.78 (2.15, 8.49) | 1.86 (1.68,2.05) |
| East Asia | 3569.55 (1228.79, 7078.77) | 17560 (7125.64, 32078.31) | 0.55 (0.2, 1.11) | 0.9 (0.36, 1.63) | 4.72 (4.63,4.81) |
| Eastern Europe | 448.14 (242.96, 677.8) | 1360.89 (779.97, 1913.58) | 0.17 (0.09, 0.25) | 0.39 (0.23, 0.54) | 3.91 (3.25,4.57) |
| Eastern Sub-Saharan Africa | 806.84 (349.31, 1517.63) | 2696.01 (1308.46, 4578.33) | 1.29 (0.55, 2.44) | 1.95 (0.93, 3.31) | 1.02 (0.78,1.26) |
| High-income Asia Pacific | 745.66 (360.52, 1251.96) | 3330.13 (1462.84, 5748.25) | 0.43 (0.2, 0.73) | 0.5 (0.23, 0.87) | 5.09 (4.77,5.41) |
| High-income North America | 5379.37 (2545.74, 8416.68) | 33841.33 (17216.62, 46918.41) | 1.48 (0.71, 2.29) | 4.74 (2.47, 6.44) | 5.63 (5.42,5.85) |
| North Africa and Middle East | 5832.53 (2744.35, 9920.76) | 28532.17 (15682.1, 39374.29) | 4.33 (2, 7.4) | 7.66 (4.02, 10.79) | 3.46 (3.25,3.66) |
| Oceania | 13.22 (5.05, 26.57) | 60.25 (27.2, 108.94) | 0.55 (0.22, 1.07) | 0.96 (0.44, 1.66) | 2.43 (2.30,2.56) |
| South Asia | 2006.67 (854.73, 3555.25) | 11762.68 (5635.29, 19262.38) | 0.38 (0.17, 0.66) | 0.86 (0.41, 1.39) | 4.05 (3.97,4.13) |
| Southeast Asia | 2484.74 (951.88, 4668.19) | 14669.81 (5986.76, 26506.95) | 1.05 (0.4, 1.98) | 2.43 (0.99, 4.37) | 4.55 (4.46,4.63) |
| Southern Latin America | 1534.58 (699.4, 2345.98) | 3833.65 (2093.49, 5466.58) | 3.59 (1.64, 5.51) | 4.2 (2.29, 5.93) | 2.17 (1.81,2.52) |
| Southern Sub-Saharan Africa | 552.78 (270.86, 892.98) | 3414.98 (1870.39, 5081.13) | 2.3 (1.11, 3.74) | 7.08 (3.71, 10.66) | 4.20 (3.71,4.69) |
| Tropical Latin America | 2030.6 (1024.02, 3075) | 9113.09 (4899.91, 12908.85) | 2.48 (1.24, 3.81) | 3.66 (1.98, 5.21) | 3.71 (3.56,3.86) |
| Western Europe | 3827.37 (2110.35, 6158.22) | 15494.23 (7633.88, 23419.64) | 0.65 (0.35, 1.02) | 1.19 (0.6, 1.79) | 4.96 (4.76,5.16) |
| Western Sub-Saharan Africa | 2006.84 (841.27, 3507.01) | 7923.37 (3786.96, 12902.8) | 2.78 (1.16, 4.79) | 5.05 (2.37, 8.15) | 1.19 (1.07,1.32) |

HTN-related CKD, chronic kidney disease due to hypertension; No, Number; ASMR, age-standardized mortality rate; EAPC, estimated annual percentage change; UI, uncertainty interval; CI, confidence interval; SDI, socio-demographic index; GBD, Global Burden of Disease.
