# Supplementary material for: Global burden of chronic kidney disease attributable to hypertension due to high body mass index: projections to 2050
Source: Ren Fail. 2026 Nov 2;48(1):2751889. doi: 10.1080/0886022X.2026.2751889 (PMC13729631; doi:10.1080/0886022X.2026.2751889)
Supplement: Supplementary file 2 [file IRNF_A_2751889_SM4227.docx]

**Table 2** DALYs and ASDR of HTN-related CKD burden attributable to High body-mass index in 1990 and 2021 and the temporal trends from 1990 to 2021.

| Characteristics | DALY cases, No. (95% *UI*) | | | ASDR per 100,000 people (95% *UI*) | | EAPC of ASDR, % (95% *CI*) |
| --- | --- | --- | --- | --- | --- | --- |
|  | 1990 | 2021 | | 1990 | 2021 | 1990–2021 |
| **Global** | 1008372.18 (468964.08, 1610433.02) | | 4257037.41 (2119854.03, 6136203.07) | 26.04 (12.18, 41.46) | 50.11 (25, 72.49) | 3.63 (3.54,3.71) |
| **Sex** |  | |  |  |  |  |
| Male | 514524.71 (235950.89, 864141.27) | | 2213202.44 (1082657.69, 3225397.84) | 29.38 (13.51, 48.6) | 56.64 (27.9, 82.48) | 3.73 (3.65,3.81) |
| Female | 493847.47 (234468.77, 757913.82) | | 2043834.97 (1032282.95, 2932755.61) | 23.64 (11.24, 36.32) | 44.76 (22.54, 64.07) | 3.52 (3.43,3.62) |
| **SDI** |  | |  |  |  |  |
| Low SDI | 75789.95 (33397.07, 131329.61) | | 267387.21 (131391.1, 449180.03) | 32.4 (14.58, 55.39) | 49.19 (23.82, 82.87) | 1.37 (1.21,1.53) |
| Low-middle SDI | 180271.57 (82101.7, 299057.41) | | 881403.24 (455361.81, 1285124.55) | 28.87 (13.44, 47.91) | 59.68 (30.18, 86.75) | 3.65 (3.59,3.70) |
| Middle SDI | 313078.78 (138517.06, 507807.57) | | 1484742.27 (719475.68, 2261097.96) | 30.23 (13.51, 49.56) | 55.79 (26.83, 84.4) | 4.06 (3.89,4.24) |
| High-middle SDI | 200214.67 (97806.88, 313072.33) | | 616990.69 (309421.78, 896716.34) | 21.43 (10.52, 33.65) | 32.26 (16.2, 47) | 3.19 (3.13,3.26) |
| High SDI | 237694.06 (120680.45, 353158.8) | | 1002355.6 (541756.46, 1309328.09) | 22.05 (11.13, 32.55) | 48.9 (26.93, 61.49) | 4.49 (4.33,4.65) |
| **GBD region** |  | |  |  |  |  |
| Andean Latin America | 17446.37 (7494.63, 27054.17) | | 85415.88 (43749.27, 118589.55) | 82.54 (35.06, 129.24) | 143.88 (72.81, 201.59) | 3.51 (3.13,3.90) |
| Australasia | 3378.15 (1940.12, 4838.19) | | 14736.44 (8773.36, 19849.75) | 15.06 (8.61, 21.62) | 24.95 (14.93, 33.25) | 3.83 (3.51,4.16) |
| Caribbean | 14043.32 (6881.8, 20964.01) | | 51939.35 (28797.03, 72531.22) | 52.96 (25.9, 79.72) | 97.26 (54.08, 135.36) | 3.76 (3.63,3.89) |
| Central Asia | 6576.35 (3662.58, 9543.46) | | 17017.01 (10105.8, 23269.65) | 13.43 (7.37, 19.58) | 20.01 (12.07, 27.55) | 1.91 (1.72,2.10) |
| Central Europe | 32711.51 (18559.64, 46047.59) | | 54442.71 (31772.13, 73233.63) | 22.97 (13.26, 32.38) | 25.07 (14.57, 33.23) | 2.68 (2.43,2.92) |
| Central Latin America | 62345.76 (33400.09, 86758.14) | | 366567.06 (213251.1, 491300.56) | 73.26 (38.43, 104.79) | 144.13 (82.98, 193.64) | 4.80 (4.29,5.31) |
| Central Sub-Saharan Africa | 13120.7 (6003.72, 23301.84) | | 61087.65 (28577.6, 101454.96) | 57.38 (26.38, 100.35) | 105.75 (49.47, 177.66) | 1.86 (1.68,2.04) |
| East Asia | 106470.47 (38171.77, 212196.28) | | 443059.48 (172944.4, 807210.71) | 12.92 (4.63, 25.5) | 21.04 (8.39, 38.26) | 4.21 (4.11,4.32) |
| Eastern Europe | 26701.49 (14366.17, 39721.45) | | 48410.97 (28289.37, 65798.04) | 10.23 (5.51, 15.11) | 14.52 (8.51, 19.63) | 2.00 (1.77,2.24) |
| Eastern Sub-Saharan Africa | 22990.27 (9761.59, 42652.6) | | 75567.38 (37175.81, 126419.45) | 29.76 (12.74, 55.37) | 42.48 (20.62, 71.33) | 0.97 (0.75,1.19) |
| High-income Asia Pacific | 19027.71 (9271.01, 32462.94) | | 56488.71 (26937.63, 94756.47) | 9.9 (4.84, 16.84) | 11.28 (5.4, 18.34) | 3.71 (3.46,3.96) |
| High-income North America | 121794.35 (57609.52, 177879.25) | | 629638.75 (333097.37, 805790.71) | 34.99 (16.57, 50.42) | 98.39 (53.92, 123.08) | 4.91 (4.71,5.11) |
| North Africa and Middle East | 148592.34 (71439.07, 244857.21) | | 672825.52 (386498.09, 899597.24) | 90.41 (43.2, 150.62) | 152 (86.25, 207.52) | 3.13 (2.97,3.29) |
| Oceania | 517.78 (199.84, 981.83) | | 2221.25 (990.9, 3900.02) | 16.67 (6.55, 31.78) | 27.43 (12.14, 47.78) | 2.27 (2.16,2.39) |
| South Asia | 76336.91 (32834.43, 136285.34) | | 417553.19 (199451.58, 681501.52) | 11.85 (5.15, 20.99) | 26.46 (12.61, 42.87) | 3.95 (3.92,3.97) |
| Southeast Asia | 78111.44 (30280.29, 147750.44) | | 416949.97 (165282.67, 766467.71) | 27.87 (10.82, 52.31) | 60.87 (24.49, 109.89) | 4.26 (4.17,4.34) |
| Southern Latin America | 33621.82 (15513.9, 49624.5) | | 70494.32 (39159.39, 95611.95) | 74.3 (34.16, 109.47) | 80.14 (44.39, 108.33) | 1.58 (1.29,1.87) |
| Southern Sub-Saharan Africa | 16709.67 (8093.48, 26253.72) | | 90219.43 (48723.11, 131500.41) | 57.26 (27.61, 89.25) | 154.33 (84.16, 223.95) | 3.77 (3.33,4.22) |
| Tropical Latin America | 63310.68 (31959.7, 92310.91) | | 218339.67 (120117.66, 297698.13) | 65.06 (32.87, 95.82) | 84.91 (46.67, 115.7) | 2.70 (2.56,2.84) |

HTN-related CKD, chronic kidney disease due to hypertension; No, Number; ASDR, Age-standardized DALY rate; DALYs, disability-adjusted life years; EAPC, estimated annual percentage change; UI, uncertainty interval; CI, confidence interval; SDI, socio-demographic index; GBD, Global Burden of Disease.
