# Supplementary material for: Global burden of chronic kidney disease attributable to hypertension due to high body mass index: projections to 2050
Source: Ren Fail. 2026 Nov 2;48(1):2751889. doi: 10.1080/0886022X.2026.2751889 (PMC13729631; doi:10.1080/0886022X.2026.2751889)
Supplement: Supplementary file 3 [file IRNF_A_2751889_SM4228.docx]

**Table S3** Decomposition analysis of changes in HTN-related CKD attributable to high BMI

| Location | Measure | Overall.difference | Aging | Population | Rate-change component | Absolute change, 1990 - 2021 |
| --- | --- | --- | --- | --- | --- | --- |
| **Global** | Deaths | 849607.02 | 211014.306 (24.84%) | 227897.22 (26.82%) | 410695.491 (48.34%) | 143311.97 |
| Male | Deaths | 484101.04 | 127996.096 (26.44%) | 130447.832 (26.95%) | 225657.115 (46.61%) | 71136.34 |
| Female | Deaths | 394007.92 | 94467.861 (23.98%) | 106702.147 (27.08%) | 192837.911 (48.94%) | 72175.64 |
| **Global** | DALYs | 11148369.7 | 2346094.132 (21.04%) | 3333528.986 (29.9%) | 5468746.577 (49.05%) | 3248665 |
| Male | DALYs | 6355627.28 | 1407767.192 (22.15%) | 1891031.523 (29.75%) | 3056828.564 (48.1%) | 1698678 |
| Female | DALYs | 5085643.54 | 1055203.586 (20.75%) | 1540752.638 (30.3%) | 2489687.317 (48.96%) | 1549987 |
| **High SDI** | Deaths | 237488.64 | 38028.227 (16.01%) | 41675.951 (17.55%) | 157784.461 (66.44%) | 43126.747 |
| **High-middle SDI** | Deaths | 204036.16 | 62459.719 (30.61%) | 36208.341 (17.75%) | 105368.1 (51.64%) | 21059.593 |
| **Middle SDI** | Deaths | 310419.09 | 108448.991 (34.94%) | 78629.964 (25.33%) | 123340.131 (39.73%) | 47086.562 |
| **Low-middle SDI** | Deaths | 351633.33 | -18152.276 (-5.16%) | 156565.531 (44.53%) | 213220.071 (60.64%) | 25485.512 |
| **Low SDI** | Deaths | 42964.53 | 569.279 (1.32%) | 25861.399 (60.19%) | 16533.852 (38.48%) | 6426.991 |
| **High SDI** | DALYs | 2507778.25 | 297412.894 (11.86%) | 501692.036 (20.01%) | 1708673.321 (68.13%) | 764661.5 |
| **High-middle SDI** | DALYs | 2120961.66 | 580079.199 (27.35%) | 449288.129 (21.18%) | 1091594.328 (51.47%) | 416776 |
| **Middle SDI** | DALYs | 4131429.23 | 1135590.243 (27.49%) | 1153897.859 (27.93%) | 1841941.133 (44.58%) | 1171663.5 |
| **Low-middle SDI** | DALYs | 2702491.88 | 399943.769 (14.8%) | 961825.229 (35.59%) | 1340722.878 (49.61%) | 701131.7 |
| **Low SDI** | DALYs | 800667.36 | 25969.899 (3.24%) | 487482.574 (60.88%) | 287214.886 (35.87%) | 191597.3 |
