# Supplementary material for: Global burden of chronic kidney disease attributable to hypertension due to high body mass index: projections to 2050
Source: Ren Fail. 2026 Nov 2;48(1):2751889. doi: 10.1080/0886022X.2026.2751889 (PMC13729631; doi:10.1080/0886022X.2026.2751889)
Supplement: Supplementary file 4 [file IRNF_A_2751889_SM4237.docx]

**Table S4** Regional patterns of HTN-related CKD attributable to high BMI and policy-relevant strategies

| **Region group** | **2021 burden pattern for HTN-related CKD attributable to high BMI** | **Key drivers** | **Suggested interventions** | **Expected impact** | **Key message for Discussion / Conclusion** |
| --- | --- | --- | --- | --- | --- |
| **Global** | Deaths and DALYs increased ≈4-fold and ≈3-fold from 1990–2021; ASMR and ASDR nearly doubled and are projected to keep rising to 2050. | High BMI accounts for ≈40% of HTN-related CKD deaths and DALYs; higher burden in men, but older women have substantial absolute burden | • Integrate weight management into HTN care (diet, physical activity, anti-obesity pharmacotherapy where feasible).  • Universal HTN screening and control in adults, prioritizing those with high BMI.  • Standardized CKD screening (eGFR + albuminuria) in obese hypertensive adults. | Coordinated implementation is expected to slow the global EAPC of ASMR/ASDR, flatten projected 2050 curves, and yield moderate DALY reductions by 2030 and larger reductions thereafter through prevention of CKD onset and progression. | Our findings underscore that high BMI has become a major global driver of HTN-related CKD, and that integrating obesity management, blood pressure control and routine CKD screening into a single care pathway is critical to reverse the currently rising mortality and DALY trends. |
| **High-income North America** | Among the fastest-rising regions (highest EAPC in ASMR and ASDR); ASDR around 2–3× the global average. | Long-standing obesity epidemic; large high-BMI-attributable fraction; ageing populations; good CKD treatment access but late detection still common. | • Fiscal/food-environment policies (SSB tax, front-of-pack labelling, restrictions on ultra-processed foods).  • Intensive weight-loss programs in primary care and HTN clinics; inclusion of GLP-1/SGLT2 agents where indicated.  • Opportunistic CKD screening in all obese hypertensive adults (including in cardiology and endocrinology clinics).  • Digital BP monitoring programs and pharmacist-led HTN management. | Strong, upstream obesity policies plus integrated HTN/CKD care could convert current steep increases into plateauing trends by ~2035 and produce substantial DALY reductions, especially via fewer patients progressing to dialysis and ESKD. | In high-income North America, where the high-BMI-attributable fraction is particularly large, our results suggest that only aggressive, upstream obesity prevention combined with intensified HTN and CKD care will be sufficient to bend the currently steeply rising ASMR and ASDR curves. |
| **High-income Asia Pacific** | Lowest ASDR worldwide, but clear positive EAPC (burden rising from a low baseline). | Rapid nutrition transition; increasing obesity, especially in urban areas; ageing societies; good health systems but variable primary prevention. | • Early, population-wide obesity prevention (school-based programs, healthy urban design, workplace wellness).  • Maintain high HTN detection and treat-to-target programs, with stronger focus on high-BMI groups.  • Public campaigns on CKD awareness and self-monitoring of BP and weight. | Early, aggressive prevention is likely to keep ASMR/ASDR at low levels and avoid convergence toward high-burden regions, thus preventing a large future DALY increase. | High-income Asia Pacific currently enjoys the lowest ASDR, and our projections indicate that prompt reinforcement of obesity and HTN prevention could preserve this advantage and prevent convergence toward the high-burden trajectories observed in Western regions. |
| **Western Europe** | Moderate ASMR/ASDR with steady increases over 1990–2021. | Rising overweight/obesity; good HTN control in many countries but residual uncontrolled BP in high-BMI men; strong primary-care infrastructure. | • Scale up salt-reduction and heart-healthy dietary policies (reformulation, public procurement standards).  • Embed systematic CKD screening for obese hypertensive adults into primary-care quality indicators.  • Expand cardiac–renal–metabolic (CRM) integrated clinics for multi-morbidity management. | Likely to bend ASMR/ASDR trends downward in the medium term as high-risk patients are identified earlier and receive reno-protective therapies; moderate DALY reductions expected by reducing progression to advanced CKD. | In Western Europe, the combination of rising high BMI and otherwise relatively strong HTN and CKD services suggests a window of opportunity, where embedding obesity and CKD management into primary-care quality metrics could translate into measurable DALY reductions. |
| **Central & Eastern Europe, Central Asia** | Low–moderate ASMR/ASDR with modest but persistent increases; Central Asia has among the lowest ASMR values. | Mixed cardiometabolic risk profiles; variable HTN control; health-system resources improving but uneven. | • Strengthen population HTN screening (workplace, community) with simplified treatment protocols.  • Promote weight-management programs focusing on diet quality and physical activity in mid-life adults.  • Ensure access to essential reno-protective drugs (RAAS blockers, SGLT2 inhibitors where feasible). | Better HTN and weight control should stabilize ASMR/ASDR and limit future increases, leading to gradual DALY reductions, particularly in younger and middle-aged adults. | In Central and Eastern Europe and Central Asia, our analysis points to modest but preventable increases in high BMI–attributable HTN-related CKD, highlighting the need for affordable HTN and weight-control packages to avoid a locked-in escalation of DALYs. |
| **East Asia** | ASMR and ASDR increased significantly (EAPC >4%); current burden is moderate but rising. | Rapid economic transition; dietary westernization; growing obesity and HTN prevalence; large ageing population. | • National salt-reduction and healthy-diet initiatives targeting traditional and processed foods.  • Target HTN screening in high-BMI urban populations and those with diabetes.  • CKD education in primary care with routine eGFR and albuminuria testing for obese hypertensive adults. | Combined risk-factor control is expected to slow the EAPC of ASMR/ASDR, especially if obesity growth is curbed, preventing a large projected DALY increase by 2050. | The rapid rise in East Asia suggests that without decisive action on diet, obesity and HTN, the region may soon face a substantial CKD burden, but our projections indicate that comprehensive risk-factor control could still avert a large proportion of future DALYs. |
| **South Asia** | ASMR and ASDR currently below global average but more than doubled since 1990. | Double burden of infectious and non-communicable disease; rapidly rising obesity in urban and peri-urban areas; low HTN awareness and control. | • Community-based HTN screening and treatment delivered via primary care and community health workers with simplified regimens.  • Low-cost lifestyle programs (salt reduction, healthy cooking, physical-activity promotion) for high-BMI adults.  • Basic CKD awareness campaigns (radio, SMS, community meetings) to encourage BP checks and early care-seeking. | Even low-cost, scalable interventions can substantially slow growth in ASDR, especially by preventing early CKD progression, yielding meaningful DALY reductions in working-age adults. | For South Asia, our findings emphasize that scalable, low-cost strategies to improve HTN awareness and weight control in rapidly urbanizing populations are essential to prevent a sharp escalation of high BMI–attributable HTN-related CKD DALYs. |
| **Southeast Asia** | Low proportion of HTN-related CKD attributable to high BMI (≈17–18% of deaths and DALYs, lowest globally) but ASMR/ASDR still increasing. | Obesity rising but still lower than in high-income regions; other risks (e.g., high sodium, infections, diabetes) important; resource constraints in some settings. | • Maintain traditional healthy dietary patterns while preventing growth of obesity (regulate marketing of ultra-processed foods, SSBs).  • HTN detection in primary care and workplaces, with special attention to urban high-BMI groups.  • Integrate CKD messages into broader NCD programs (diabetes, CVD). | Targeted prevention of obesity at an early stage is likely to keep the BMI-attributable fraction low and prevent a steep rise in ASMR/ASDR, limiting future DALY growth. | The relatively low high-BMI-attributable fraction in Southeast Asia suggests a critical prevention window, where preserving traditional diets and containing obesity could prevent the region from replicating the high ASDR seen elsewhere. |
